# Pregnancy in Women Receiving Maintenance Dialysis

**DOI:** 10.1016/j.xkme.2024.100950

**Published:** 2024-12-19

**Authors:** Cristina Popa, Priyadarshini John, Prasoon Verma, Sehrish Ali, Silvi Shah

**Affiliations:** 1 Department of Internal Medicine—Nephrology, University of Medicine and Pharmacy “Grigore T Popa”, Iasi, Romania; 2 Department of Nephrology, Osmania General Hospital, Hyderabad, India; 3 Department of Pediatrics, University of Cincinnati College of Medicine, Cincinnati, OH; 4 Division of Neonatology, Cincinnati Children’s Hospital Medical Center, Cincinnati, OH; 5 Division of Nephrology, Baylor College of Medicine, Houston, TX; 6 Division of Nephrology, University of Cincinnati College of Medicine, Cincinnati, OH

**Keywords:** Dialysis, pregnancy, outcomes, end-stage kidney disease, management

## Abstract

Women with kidney failure experience pathophysiological changes that frequently result in disruption of the hypothalamic-pituitary-ovarian axis. Because of these hormonal disturbances, women with kidney disease often experience oligomenorrhea, amenorrhea, sexual dysfunction, and infertility. Preconception counseling, partnered with the early identification and optimal management of risk factors, such as hypertension and discontinuation of teratogenic medications, should be pursued for females contemplating conception. Pregnancy in women receiving maintenance dialysis is associated with a high risk of adverse maternal and fetal outcomes and should be managed by a multidisciplinary team of providers. In this review article, we discuss pregnancy incidence, pregnancy outcomes, and management of pregnancy among women receiving maintenance dialysis.

In 1947, the first case report of peritoneal dialysis in a patient with eclampsia was published by Joan Marsden.^1^ This marked the beginning of a new era in the management of pregnancy in women with kidney disease. However, nearly 30 years later, in 1975, *The Lancet* still noted that the children of women with kidney disease used to be born dangerously or not at all—not at all if their doctors had their way, highlighting the struggles of women with kidney disease and the approach of the medical community to pregnancy in this population.^2^ Today, outcomes among pregnant women with kidney failure have improved through a better understanding of the physiology and pathophysiology of pregnancy in this population, increased research and awareness, and improved treatment by intensification of dialysis and multidisciplinary care. In this review, we summarize the current understanding of the pathophysiology behind the differences in reproductive health seen among patients receiving maintenance dialysis and the incidence, outcomes, and management of pregnancy in this population.

### Fertility and Kidney Failure

Women with kidney failure experience disruption of the hypothalamic-gonadal axis that leads to irregularity in the menstrual cycle, anovulation, decreased libido, and impaired fertility. Women on maintenance dialysis, especially with amenorrhea, have high serum prolactin because of impaired kidney clearance, increased luteinizing hormone and follicular stimulating hormone, and decreased estradiol and progesterone concentration. The persistently elevated gonadotropins because of loss of negative feedback on hypothalamic and pituitary centers and absence of luteinizing hormone surge leads to anovulation.^3^^,^^4^ Of importance, there is restoration of hormones such as gonadotropins and prolactin after kidney transplantation, suggesting that the underlying cause of the hormonal imbalances with kidney failure is related to the dysfunction of the kidneys rather than permanent damage to the hypothalamic-pituitary-ovarian axis.^5^^,^^6^

There is a high prevalence of menstrual abnormalities in women on maintenance dialysis, specifically irregular menses (47%) and amenorrhea (41%).^7^ Lin et al^6^ performed a cross-sectional study that showed a high prevalence of menstrual disturbances by kidney replacement therapy: nocturnal hemodialysis (55%), continuous ambulatory peritoneal dialysis (72.1%), and hemodialysis (76.1%).

The prevalence of sexual dysfunction is high among women on maintenance dialysis and is caused by hormonal derangements in addition to psychosocial factors, such as anxiety, depression, and poor social and body image.^8^^,^^9^ Hormonal changes may lead to dry skin, increased wrinkles, urinary incontinence, hot flashes, vaginal dryness, and atrophy, which may be associated with sexual dysfunction.^10^ In the population treated with dialysis, negative body image from feeling bloated, having edema, dialysis fistulas, catheters, or equipment in the bedroom has been listed as a factor contributing to decreased sexual satisfaction and desire.^11^ Women are less content with their physical appearance than men and have a more negative impression of their bodies, particularly regarding sexual activity.^12^

### Incidence of Pregnancy Among Women Receiving Maintenance Dialysis

The pregnancy rate among women aged 15-44 years receiving dialysis is ∼18 per 1,000 person-years in the United States, with the highest rate being observed in women aged 20-24 at ∼41 pregnancies per 1,000 person-years.^13^ Women treated with peritoneal dialysis have a 53% lower likelihood of pregnancy than those treated with hemodialysis. The reasons for the lower rates of conception among women receiving peritoneal dialysis remain unclear, although it is hypothesized that hypertonic dextrose solutions and the presence of fluid in the peritoneal cavity may interfere with ovum transit to the uterus. Women with kidney failure because of malignancy, glomerulonephritis, hypertension, and secondary glomerulonephritis/vasculitis have a higher pregnancy rate than women with kidney failure because of diabetes.^13^

### Pregnancy Outcomes Receiving Maintenance Dialysis

Pregnancy among women receiving maintenance dialysis is associated with a higher risk of adverse outcomes. The rate of premature birth is much higher among pregnant women receiving hemodialysis when compared with the general population, with a 2016 meta-analysis by Piccoli et al^14^ noting that 88% of births were preterm among patients treated with dialysis. Another the Australia and New Zealand Dialysis and Transplant Registry study also reported that, between 1991 and 2013, premature births among pregnant women receiving hemodialysis were quite high, at ∼80%.^15^ More recently, a United States registry study that included >300 pregnant hemodialysis women reported that 45% had preterm births.^16^ As the incidence of preterm delivery may have changed over time, it is crucial to consider the era of the source data when considering the incidence of preterm delivery. Pregnant women receiving hemodialysis have a higher rate of cesarean section, >60%, and poorer peripartum outcomes, including a higher rate of blood transfusions, longer hospital stays with more than half of the patients staying >6 days in the hospital, and a higher risk of sepsis, which occurs in 6% of deliveries.^16^

The rate of live births is lower, and the perinatal mortality is higher among women undergoing dialysis. Using United Renal Data System Data, Shah et al^13^ reported that live births occurred in 27% of pregnancies, whereas 3% of pregnancies resulted in stillbirth, 29% resulted in spontaneous abortion, 8% of pregnancies had therapeutic abortion, 3% had ectopic/trophoblastic pregnancies, and ∼one-third of pregnancies had unknown outcomes. The Australia and New Zealand Dialysis and Transplant Registry showed the perinatal death rate was higher in the dialysis cohorts (162 per 1,000 births) than in the nondialysis cohort defined by women who did not receive any kidney replacement therapy (9 per 1,000 births).^15^ Another study reported a perinatal mortality rate of 2.6% for pregnancies among women receiving maintenance hemodialysis.^16^ In addition, babies exposed to maintenance dialysis had higher odds of prematurity, small for gestational age status, poor birth condition, need for resuscitation at birth, neonatal intensive care admission, and longer duration of hospitalization. Moreover, preterm babies of mothers treated with dialysis had higher odds of adverse outcomes, including an 11-fold higher risk of birth weight lower than 2,500 gm.^15^

### Management of Pregnancies on Maintenance Dialysis

Managing a pregnant patient receiving maintenance dialysis can be challenging because of the higher risk for maternal and fetal complications, such as preeclampsia, fetal growth restriction, preterm delivery, stillbirths, and neonatal deaths.^17^^,^^18^ A multidisciplinary team consisting of the obstetrician, the nephrologist, a dietitian, and a maternal-fetal medicine physician should be engaged before, during, and after pregnancy.

#### Preconception Counseling and Contraception

Preconception counseling should include a discussion about the risks and benefits of pregnancy for all women of childbearing age who are receiving maintenance dialysis. Kidney transplantation should be encouraged, as reproductive function improves after kidney transplantation, and pregnancy postkidney transplantation is associated with fewer maternal and fetal complications.^19^^,^^20^

All women of childbearing age who are sexually active and do not wish to become pregnant should be advised to be on contraception. Irregular menstrual cycles in this population make it difficult to rely solely on natural family planning methods to avoid conception. Although this discussion should be held by both gynecologists and nephrologists, it is often overlooked by nephrologists. A survey performed in the United States by Sachdeva et al^21^ showed that only 46% of nephrologists or their staff members counseled childbearing women about contraception. There is limited education and training on how to discuss and address reproductive health for women with kidney failure, which may result in a lack of confidence. Consequently, contraceptive use in female patients on maintenance dialysis is low. A retrospective cohort study evaluated contraceptive use in >35,000 women receiving dialysis included in the United States Renal Data System and reported it to be only 5.3%.^22^ Increased education and knowledge are needed in the community to encourage more robust conversations with health care providers regarding family planning, leading to shared decision-making and ultimately improving care.

Contraceptive methods include agents containing estrogen and progesterone (pill, ring, and patch), progesterone-only agents (injection and pill), intrauterine devices, barrier methods, and sterilization. Figure 1 outlines the advantages and disadvantages associated with various contraceptive options. Oral contraceptive agents containing estrogen/progestin are commonly recommended to women of childbearing age as an effective birth control method and are associated with benefits, such as protection against ovarian cancer^23^^,^^24^ and endometrial cancer.^25^ On the contrary, combined hormonal contraceptives are associated with an increased risk of hypertension, cervical cancer, breast cancer, and ischemic stroke.^25^, ^26^, ^27^ Combined hormonal contraceptives, containing both estrogen and progesterone, should be used with caution in patients receiving dialysis with uncontrolled hypertension or systemic lupus with positive antiphospholipid antibodies, because estrogen-containing contraceptives are associated with increased cardiovascular and thrombotic risks.^28^, ^29^, ^30^

**Figure 1:**
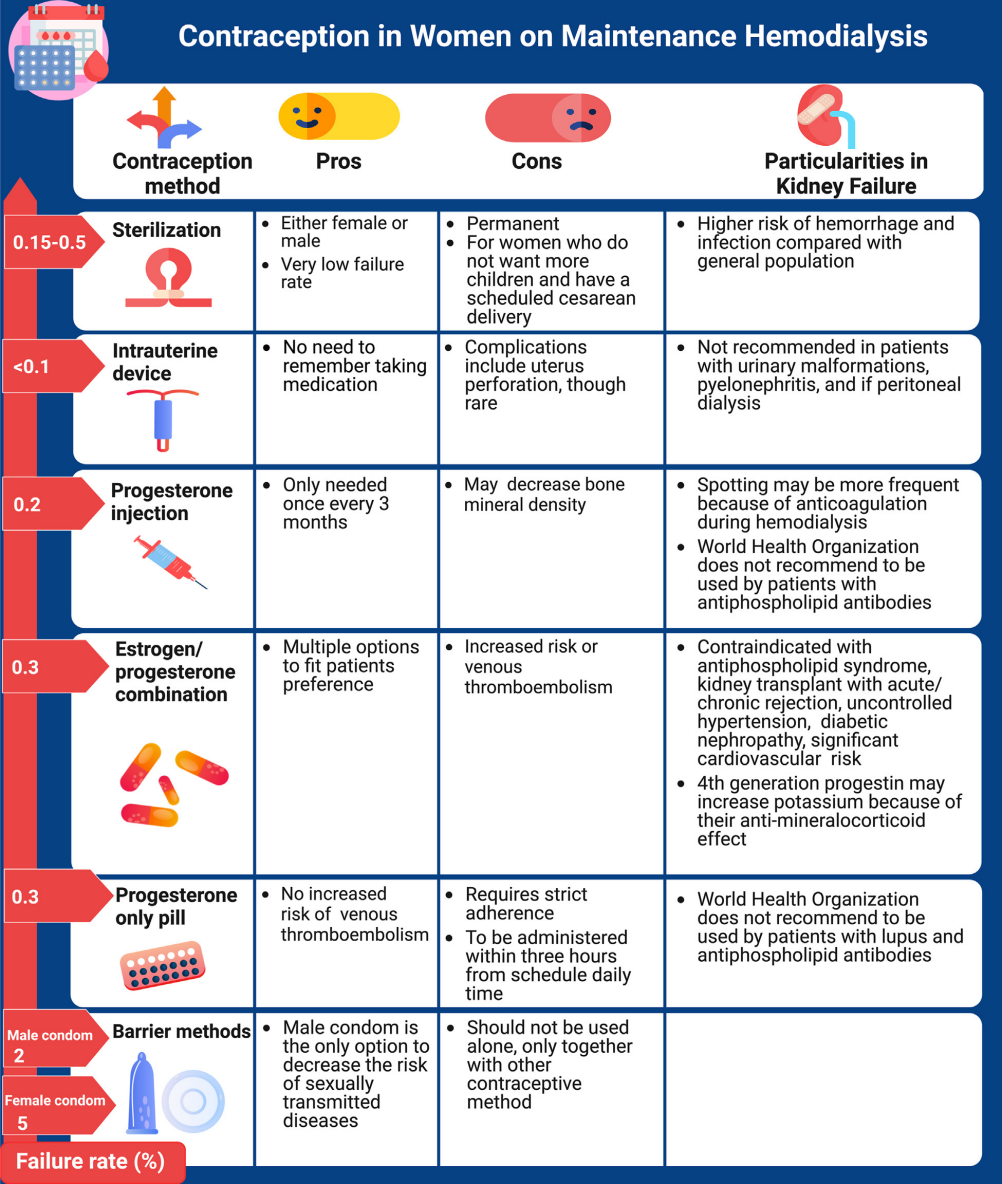
Contraception options for women receiving maintenance dialysis.

Progestin-only contraceptives may be used as an alternative as they have not been linked with worsening hypertension or ovarian, endometrial, and colorectal cancers.^17^^,^^23^^,^^31^ In comparison to combined hormonal contraceptives, progestin-only pills have a lower cardiovascular risk.^32^ The fourth-generation oral progestin, drospirenone, may theoretically increase potassium levels because of its anti-mineralocorticoid activity.^33^ A meta-analysis of studies of women without kidney failure showed that the thrombotic risk was higher in women using progestin-only injections than non-users (relative ratio [RR], 2.67; 95% CI, 1.29-5.53), while progestin-only pills or intrauterine devices did not increase this risk.^34^ In the general population, long-term use of progestin-only injection, depot medroxyprogesterone acetate, has been associated with bone mass loss and osteoporosis.^35^ Intrauterine devices remain the preferred method of contraception because of low failure rates and are not associated with an increased risk of hypertension, thrombotic events, or cardiovascular events.^32^ Barrier methods, which include condoms, diaphragms, cervical caps, and sponges, are nonhormonal contraceptives and not recommended for use because of their high failure rate.^32^ Sterilization, which includes female tubal ligation and male vasectomy, is the only permanent contraceptive method available.

#### Prenatal Care

Preconception counseling on the higher risk of adverse maternal and fetal outcomes with pregnancy among women receiving maintenance dialysis is imperative. In women with autoimmune diseases, such as lupus nephritis, it is recommended to postpone conception until the disease is inactive for at least 6 months before conception to reduce the risks of adverse maternal and fetal outcomes.^36^ Low-dose aspirin should be started by the end of the first trimester for all pregnant women receiving maintenance dialysis to reduce the risk of preeclampsia.^37^ The recommended blood pressure (BP) goal during pregnancy is < 140/90 mm Hg.^38^ Folic acid is removed with hemodialysis, and the dose should be increased to 5 mg following conception.^39^

#### Anticoagulation in Pregnancy

Anticoagulation during pregnancy is recommended in patients with kidney failure with risk factors for venous thromboembolism, including nephrotic range proteinuria, hypoalbuminemia with serum albumin < 2 mg/dL, positive antiphospholipid syndrome, and previous history of thrombosis.^38^ Anticoagulation can be considered for patients with additional risk factors such as immobility and obesity and patients with higher risks of thrombosis such as membranous nephropathy and vasculitis.^40^ Current guidelines recommend unfractionated heparin or low molecular weight heparin for anticoagulation during pregnancy and 6 weeks postpartum.^38^

#### Medication Optimization

Nephrologists should thoroughly review medications during preconception counseling. Teratogenic medications should be discontinued at least 6 weeks before planned conception. If an unexpected pregnancy occurs, these medications should be discontinued immediately after the diagnosis is made. Women who have received a cumulative dose of more than 10 gm of cyclophosphamide in the past should be assessed early for infertility, and, if assisted reproduction techniques are sought, single embryo transfer should be pursued.^38^

Figure 2 outlines medications that are considered safe or contraindicated during pregnancy on maintenance dialysis. Immunosuppressive agents that are associated with teratogenicity, such as mycophenolate or cyclophosphamide, should be discontinued 6 weeks before planned conception to prevent fetal malformation and spontaneous pregnancy loss.^41^^,^^42^ Azathioprine is the first-line agent to replace mycophenolate during pregnancy. Calcineurin inhibitors are other pregnancy-safe alternative agents that can be considered for immunosuppression, because they are not associated with an increased risk of pregnancy loss or congenital malformations.^42^ However, especially tacrolimus, if unmonitored during pregnancy, can lead to complications including infection (22%), hypertension (56%), preeclampsia (32%), and low birth weight infants (46%).^43^ Evidence on the use of biological agents (infliximab, etanercept, adalimumab, and rituximab) during pregnancy is limited, and manufacturers recommend discontinuation of them before conception.^44^ Although rituximab has been associated with transient B cell depletion in mothers and infants, a recent case series did not report this.^45^

**Figure 2:**
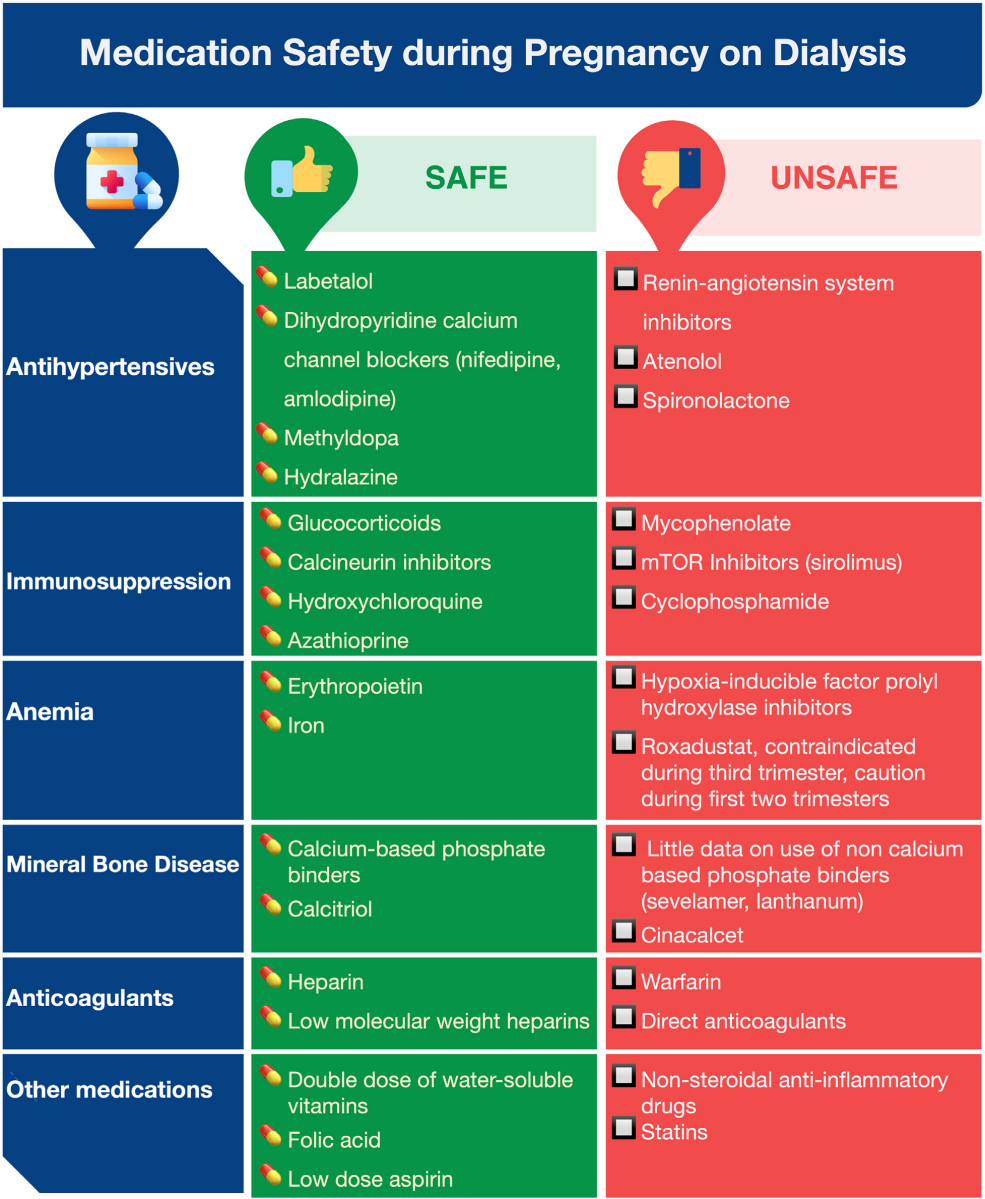
Safety of medications during pregnancy among patients receiving maintenance dialysis.

Antihypertensive drugs of choice in pregnancy include β-blockers such as labetalol, dihydropyridine calcium channel blockers such as nifedipine, hydralazine, and methyldopa. Angiotensin-converting enzyme inhibitors (ACEis) and angiotensin receptor blockers (ARBs) should be discontinued 6 weeks before conception or at the diagnosis of pregnancy because of their high risk for teratogenicity.^46^ A meta-analysis and systemic review published in 2021 showed that in comparison to those without gestational ACEi/ARB use, the use of ACEi/ARB in the first trimester resulted in a higher risk of fetal malformations (OR, 1.81; 9% CI, 1.42-2.34), cardiovascular malformations (OR, 2.50; 95% CI, 1.62-3.87), and stillbirths (OR, 1.75; 95% CI, 1.21-2.53).^47^ Second trimester and third-trimester use of ACEi/ARB is associated with complications, such as oligohydramnios and fetal and neonatal death.^48^ Diuretics can be used during pregnancy in women with kidney failure.^49^ Currently, there are no human trials to report the safety of sodium-glucose cotransporter-2 inhibitors in pregnant patients. Animal data suggest that they may affect in-utero kidney development and maturation, and it is therefore advised to stop sodium-glucose cotransporter-2 inhibitors 6 weeks before conception.^50^ Statins have been contraindicated in pregnancy because of their teratogenic risk. The risk of teratogenicity has been shown in animal studies, in which it is commonly seen with lipophilic statins such as atorvastatin and less commonly seen with hydrophilic statins such as pravastatin.^51^ In July 2021, however, the United States Food and Drug Administration requested to remove this recommendation based on recent human studies, which have failed to show major congenital malformations or stillbirth with statin use.^52^ A cohort study published in 2015 failed to show a statistically significant increase in the risk of teratogenic effects after adjusting for potential confounders.^53^ Another study did not show congenital anomalies with statin use during pregnancy but did identify a greater risk of low birth weight (RR, 1.51; 95% CI, 1.05-2.16) and premature birth (RR, 1.99; 95% CI, 1.46-2.71) in comparison with women without statin use.^54^

Anemia may worsen during pregnancy because of volume expansion and increased demands of the growing fetus.^46^ Increased doses of parenteral iron (oral or intravenous) and erythropoietin-stimulating agents may be needed during pregnancy to maintain a hemoglobin level of 10-11 g/L.^55^ For hyperphosphatemia during pregnancy, calcium-based binders and vitamin D analogs are preferred over non-calcium-based phosphate binders or calcimimetics because of limited data on the latter agents with pregnancy.^56^

#### Hypertension Management

Hypertension during pregnancy is associated with many adverse maternal and fetal outcomes, such as maternal cardiovascular complications, fetal morbidity, intrauterine growth restriction, and premature delivery.^57^ The American College of Obstetricians and Gynecologists Task Force has classified hypertension in pregnancy into 4 categories: chronic hypertension, gestational hypertension, preeclampsia, and preeclampsia superimposed on chronic hypertension.^58^ Chronic hypertension is a pre-existing diagnosis of hypertension before 20 weeks of gestation or persistent hypertension >12 weeks postpartum. Gestational hypertension is new-onset hypertension, defined as BP ≥ 140/90 mm Hg after 20 weeks of gestation without any proteinuria or end-organ. Preeclampsia is defined as new-onset hypertension after 20 weeks of gestation with proteinuria (≥300 mg/d) or end-organ dysfunction in at least 1 organ. End-organ damage can include thrombocytopenia (platelet count < 100,000/ μL,), kidney dysfunction (serum creatinine > 1.1 mg/dL or doubling of the serum creatinine concentration in the absence of other kidney disease), liver enzymes twice the upper limit of normal range, pulmonary edema, or new-onset cerebral or vision changes. Preeclampsia superimposed on chronic hypertension is new-onset proteinuria or end-organ dysfunction coupled with worsening hypertension after 20 weeks of gestation in women with chronic hypertension.^58^ In women treated with dialysis, it is challenging to diagnose preeclampsia especially among those with pre-existing hypertension because of limited utility of urine protein creatinine ratio with anuria. New onset of hypertension after 20 weeks or worsening of hypertension in women with pre-existing hypertension along with classical symptoms (headache, nausea, vomiting, epigastric pain, and visual disturbances) and laboratory abnormalities (hemolysis, thrombocytopenia, liver dysfunction, and high uric acid) of end-organ damage can be used to make a diagnosis of preeclampsia.

The risk of preeclampsia among women undergoing dialysis is high^59^ and is associated with a 1.7 to 3.6-fold increase in risk for cardiovascular mortality.^60^ Many pathophysiological mechanisms are proposed to cause preeclampsia. An imbalance of angiogenic factors, increased renin-angiotensin-aldosterone system activity, oxidative stress, immunologic dysfunction, and impaired amino acid metabolism results in abnormal placental perfusion, failure to remodel spiral arteries, and endothelial dysfunction.^61^ In the nondialysis population, kidney findings include glomerular endotheliosis and podocyturia leading to kidney dysfunction and reduced nephron mass.^62^

Two promising biomarkers, soluble fms-like tyrosine kinase 1 (sFlt-1) and placental growth factor (P1GF), are being studied in the general population to help early detection and management of preeclampsia in pregnant women at risk. During normal pregnancy, P1GF and vascular endothelial growth factor help maintain vascular homeostasis. In patients with preeclampsia, the placenta secretes sFLT1, which binds to circulating P1GF and vascular endothelial growth factor, inhibiting their action and leading to endothelial dysfunction. Measuring SFlt-1 and P1GF can be useful in managing preeclampsia. The Prediction of Short-Term Outcome in Pregnant Women with Suspected Preeclampsia Study was designed to assess the role of SFlt-1/P1GF ratio in predicting the presence or absence of preeclampsia in the short term. The study showed that SFlt-1/P1GF ratio of 38 or less has a high negative predictive value of 99.3% (95% CI, 97.9-99.9) for ruling out preeclampsia within 1 week during the second or third trimester.^63^ These studies support the role of using these biomarkers in diagnosing preeclampsia, which may minimize adverse outcomes associated with it, however, the data in the dialysis population are limited,

A multicenter randomized clinical trial in the general population, the control of hypertension in pregnancy study, compared tight BP control (diastolic BP of 85 mm Hg) to less tight BP control (diastolic BP > 100 mm Hg), and found that the tight BP group had less incidence of severe hypertension (BP > 160/110 mm Hg), but the risk of adverse pregnancy outcomes was not significant.^64^ A recent multicenter randomized clinical trial, the chronic hypertension and pregnancy trial, evaluated the effect of BP control < 140/90 mm Hg on adverse pregnancy outcomes in women in the general population at the gestational age of 23 weeks or less. Achieving a BP of < 140/90 mm Hg was associated with lower adverse pregnancy outcomes (30.2%) in comparison with the control group (37.0%) (RR, 0.82; 95% CI, 0.74-0.92).^65^ Based on these trials in the general population, we recommend to attain a BP goal of < 140/90 mm Hg during pregnancy in women on maintenance dialysis.

For patients with preeclampsia, delivery is recommended at 37 weeks or later if no severe features such as hemolysis, thrombocytopenia, or liver dysfunction are observed.^58^ Stabilization and delivery at or >34 weeks are recommended in those women with severe features or adverse maternal or fetal conditions, such as fetal growth restriction or fetal distress. If delivery is done before 34 weeks’ gestation, corticosteroids should be administered to the mother for fetal lung maturity.^58^ Many patients with preeclampsia are treated with magnesium sulfate to minimize the risk of seizures. As magnesium is excreted by kidneys, a lower dose is recommended for women with kidney failure. Careful monitoring for clinical signs of magnesium toxicity and magnesium levels is needed.^61^

#### Fetal and Maternal Monitoring during Pregnancy

Despite the best care offered to pregnant dialysis women, only 75%-80% of pregnancies reach the second trimester with surviving infants, out of which 82% are born premature.^66^ Fetal morbidity can be because of premature rupture of membranes, premature labor, and cervical incompetence.

The incidence of cervical incompetence is high in women treated with dialysis, and screening is suggested between 18 and 20 weeks. Preeclampsia should be screened by monitoring BP and laboratory chemistries, including complete blood counts and liver function tests, twice a month.^67^ Minimizing fluctuations in BP during dialysis sessions prevents uteroplacental insufficiency. For a patient receiving dialysis, pregnancy is considered high risk, and fetal anomaly screening is recommended.^68^

### Dialysis Prescription During Pregnancy

#### Peritoneal Dialysis During Pregnancy

Although pregnancy is rare in women who are receiving peritoneal dialysis because of the possible effects of hypertonic dialysate on the transit of the ovum to the uterus, women can continue pregnancy on peritoneal dialysis with modifications in peritoneal dialysis prescriptions. The advantages of peritoneal dialysis during pregnancy are controlled ultrafiltration, better hemodynamic stability, a lesser incidence of anemia, better control of BP, and avoidance of anticoagulation.

As the gestational age increases, peritoneal dialysis becomes difficult for many patients. With the increasing size of the uterus, effective peritoneal exchanges may not take place because of a reduction in effective peritoneal surface area, even though the peritoneal membrane continues to maintain the clearance and ultrafiltration capability during pregnancy.^69^ Thus, the prescription may need to be adjusted during pregnancy to reduce the dwell volume and increase the number of exchanges.^70^ A successful pregnancy occurring during peritoneal dialysis may also depend on residual kidney function. Women with residual urine output of > 500 mL have been shown to have better fetal and maternal outcomes.^71^

Similarly, the lesser the uremic milieu, the better the fetal outcomes. The following modifications in peritoneal dialysis prescriptions are suggested during pregnancy (Fig 3):

● Intensify the prescription by decreasing the dwell volume and increasing the frequency of exchanges. This may be facilitated using cyclers, particularly in the second trimester and beyond.
● Use both manual daytime exchanges and cycler-assisted overnight exchanges to facilitate adequate dialysis in the third trimester, a time during which further reduction in the dialysate volume is needed.
● Use tidal peritoneal dialysis, which leaves 15% of the dwell volume in situ while draining, to reduce drain pain and gastroesophageal reflux.
● Minimize the use of highly hypertonic peritoneal dialysate like that containing 4.25% dextrose.
● Adopt right or left lateral decubitus position during the draining phase.^72^

**Figure 3:**
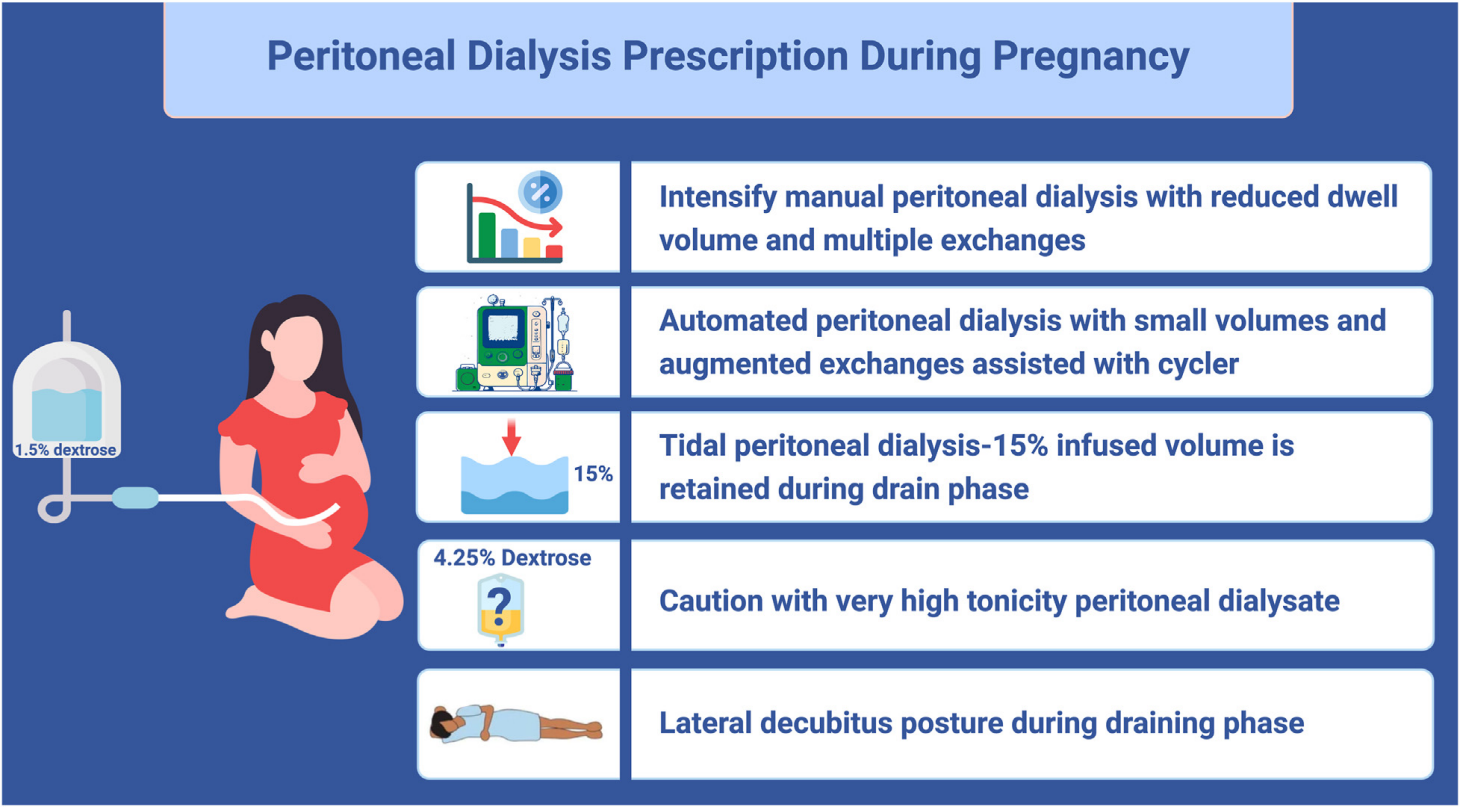
Peritoneal dialysis during pregnancy.

Although it is not a regular practice to check the adequacy of peritoneal dialysis during pregnancy, a Kt/V between 2.2 and 2.4 is recommended, which usually requires a daily dialysate volume of about 20 liters.^73^ A suggested calorie intake of 25 kcal/kg/d is recommended during pregnancy for those receiving peritoneal dialysis.^74^

Complications associated with peritoneal dialysis during pregnancy include peritonitis, exit site infections, catheter displacement, hemoperitoneum, drain pain, gastroesophageal reflux, and, in rare instances, postpartum peritonitis and traumatic uterine rupture from peritoneal catheter. Polyhydramnios (excess amniotic fluid), though rare among women receiving peritoneal dialysis, may occur if maternal urea levels are high enough to cause an osmotic diuresis in the fetus causing increased urine production by the fetus. Monitoring for electrolyte derangements such as hypokalemia and hypophosphatemia is essential to prevent arrhythmias and other fetal complications, and supplementation is warranted accordingly.^75^

#### Hemodialysis During Pregnancy

Intensive hemodialysis is recommended during pregnancy because it is associated with better fetal and maternal outcomes. Up to 36 hours of dialysis weekly is recommended for women, targeting predialysis serum blood urea nitrogen of < 50 mg/dL. In a series of 344 pregnancies, Okundaye et al^76^ observed that infants born to women who had >20 hours/wk of dialysis had prolonged gestational age and a higher birth weight. Intensive hemodialysis is associated with improved pregnancy outcomes with a lower incidence of prematurity, a higher incidence of live birth rate, higher birth weight, and lower neonatal mortality rates.^68^

To achieve a predialysis blood urea of less than 50 mg/dL, 4-6 sessions of 4-6 hours of dialysis per week are usually needed. Frequent dialysis is aimed at maintaining adequate uteroplacental flow without compromising on net fluid removal. Biocompatible and single-use dialyzers are recommended to prevent fetal toxicity secondary to reuse agents such as formaldehyde and ethylene oxide. A biocompatible and single-use dialyzer is preferred. Respiratory alkalosis with compensated metabolic acidosis is usually seen during pregnancy. Hence, dialyzing a pregnant woman would mandate low bicarbonate dialysate to avoid alkalemia. Although the data are limited, it is recommended to use a dialysate bath with 25-30 mmol/L bicarbonate during pregnancy. To minimize the risks of hypocalcemia, hypokalemia, and hypophosphatemia in the mother, it is recommended to use dialysate with a calcium concentration of 2.5 mEq/L and dialysate potassium between 3 and 3.5 mEq/L.^77^ Periodic monitoring of calcium, phosphorus, and other electrolytes is suggested for early identification and correction of electrolyte abnormalities.

Ultrafiltration during dialysis in a pregnant woman can be challenging. Fetal weight gain after the first trimester is mostly constant at 0.3-0.5 kg per week. Therefore, as long as the patient is euvolemic, the dry weight can be increased by 400 gm per week to account for the growing fetus weight of 500 gm per week.^78^ Slow continuous ultrafiltration can be considered for excessive weight gain or refractory fluid overload states during pregnancy.

The suggested calorie intake during pregnancy in women receiving hemodialysis is 35 kcal/kg/day.^74^ Protein intake of 1.5-1.8 gm/kg/day is recommended to account for adequate nitrogen balance and development of the fetus. Water-soluble vitamins should be continued throughout the pregnancy (Fig 4).

**Figure 4:**
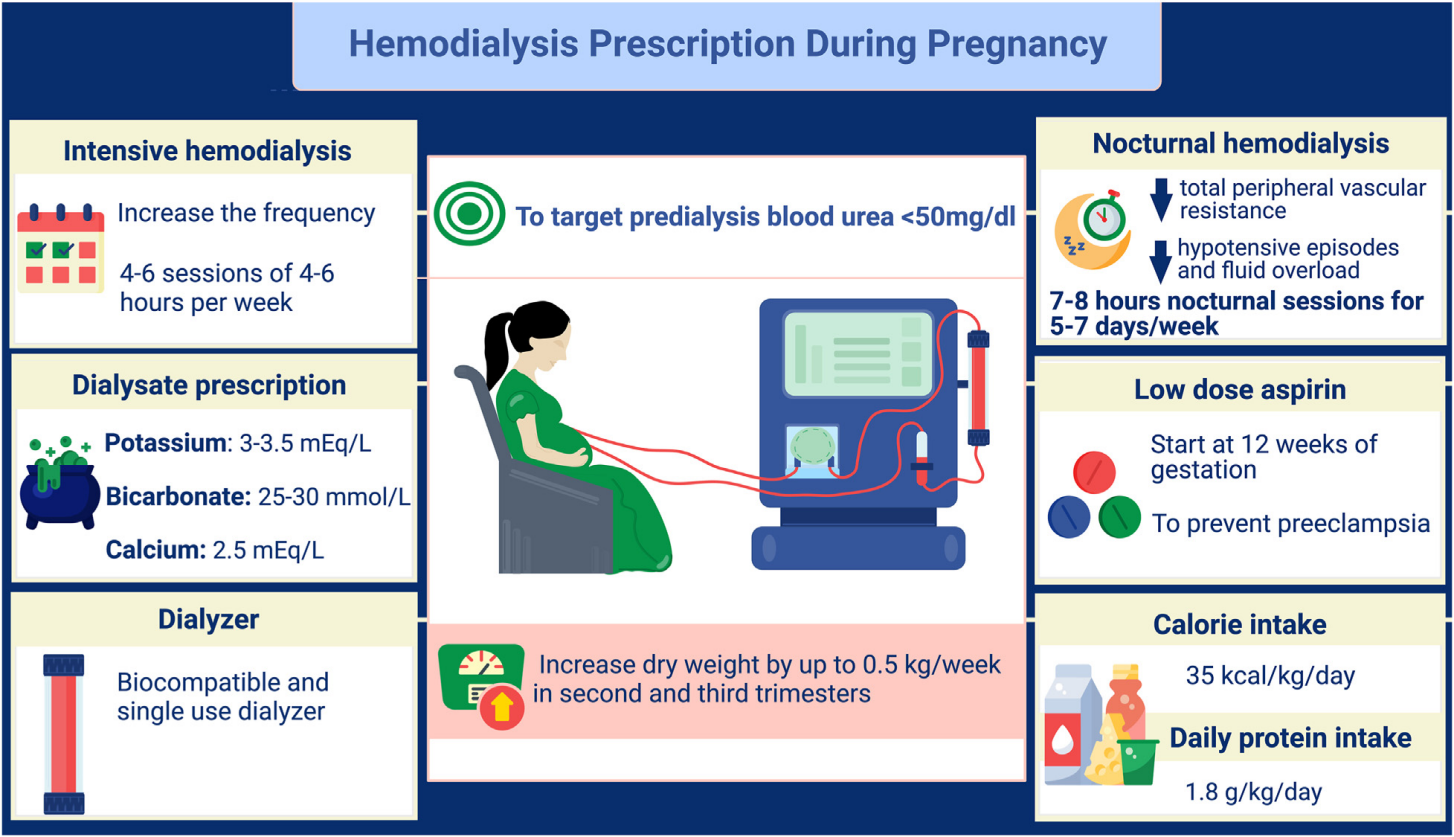
Hemodialysis during pregnancy.

Nocturnal hemodialysis in pregnant women is safe, and women who conceived while utilizing nocturnal hemodialysis as their kidney replacement therapy can continue with the modality. Women receiving nocturnal hemodialysis tend to have decreased total peripheral vascular resistance, which decreases the incidence of fluctuating BP or volume overload. Nocturnal hemodialysis prescriptions during pregnancy usually require 7-8 hour sessions, 5-7 days a week.^79^ Women performing nocturnal hemodialysis might require phosphate supplementation in the dialysate to avoid hypophosphatemia because of prolonged dialysis sessions.

## Conclusion

This review presents the current best practices in evaluating and managing pregnant women on maintenance dialysis. Women with kidney failure undergoing dialysis must be counseled regarding the increased risk of maternal and fetal complications with pregnancy. A multidisciplinary team consisting of a nephrologist, maternal-fetal medicine specialist, and neonatologist should manage pregnancy in women receiving dialysis.

## References

[1] Marsden J.A. (1947). The use of peritoneal dialysis in a case of eclampsia. Med J Aust.

[2] (1975). Pregnancy and renal disease. Lancet.

[3] Holley J.L. (2004). The hypothalamic-pituitary axis in men and women with chronic kidney disease. Adv Chronic Kidney Dis.

[4] van Eps C., Hawley C., Jeffries J. (2012). Changes in serum prolactin, sex hormones and thyroid function with alternate nightly nocturnal home haemodialysis. Nephrology (Carlton).

[5] Wang G.C., Zheng J.H., Xu L.G. (2010). Measurements of serum pituitary-gonadal hormones and investigation of sexual and reproductive functions in kidney transplant recipients. Int J Nephrol.

[6] Lin C.T., Liu X.N., Xu H.L., Sui H.Y. (2016). Menstrual disturbances in premenopausal women with end-stage renal disease: a cross-sectional study. Med Princ Pract.

[7] Rytz C.L., Kochaksaraei G.S., Skeith L. (2022). Menstrual abnormalities and reproductive lifespan in females with CKD: a systematic review and meta-analysis. Clin J Am Soc Nephrol.

[8] Theofilou P.A. (2012). Sexual functioning in chronic kidney disease: the association with depression and anxiety. Hemodial Int.

[9] Oliverio A.L., Bramham K., Hladunewich M.A. (2021). Pregnancy and CKD: advances in care and the legacy of Dr Susan Hou. Am J Kidney Dis.

[10] Ali S., Dave N.N. (2020). Sexual dysfunction in women with kidney disease. Adv Chronic Kidney Dis.

[11] Álvarez-Villarreal M., Velarde-García J.F., Chocarro-Gonzalez L., Pérez-Corrales J., Gueita-Rodriguez J., Palacios-Ceña D. (2019). Body changes and decreased sexual drive after dialysis: a qualitative study on the experiences of women at an ambulatory dialysis unit in Spain. Int J Environ Res Public Health.

[12] Lewis H., Arber S. (2015). The role of the body in end-stage kidney disease in young adults: gender, peer and intimate relationships. Chronic Illn.

[13] Shah S., Christianson A.L., Meganathan K., Leonard A.C., Schauer D.P., Thakar C.V. (2019). Racial differences and factors associated with pregnancy in ESKD patients on dialysis in the United States. J Am Soc Nephrol.

[14] Piccoli G.B., Minelli F., Versino E. (2016). Pregnancy in dialysis patients in the new millennium: a systematic review and meta-regression analysis correlating dialysis schedules and pregnancy outcomes. Nephrol Dial Transplant.

[15] Hewawasam E., Davies C.E., Li Z. (2022). Determinants of perinatal outcomes in dialyzed and transplanted women in Australia. Kidney Int Rep.

[16] Lavie A., Czuzoj-Shulman N., Spence A.R., Abenhaim H.A. (2022). Characteristics and outcomes among pregnant women with end-stage renal disease on hemodialysis. J Matern Fetal Neonatal Med.

[17] Burgner A., Hladunewich M.A. (2020). Contraception and CKD. Clin J Am Soc Nephrol.

[18] Stringer E.M., Kaseba C., Levy J. (2007). A randomized trial of the intrauterine contraceptive device vs hormonal contraception in women who are infected with the human immunodeficiency virus. Am J Obstet Gynecol.

[19] McKay D.B., Josephson M.A. (2008). Pregnancy after kidney transplantation. Clin J Am Soc Nephrol.

[20] Shah S., Venkatesan R.L., Gupta A. (2019). Pregnancy outcomes in women with kidney transplant: Metaanalysis and systematic review. BMC Nephrol.

[21] Sachdeva M., Barta V., Thakkar J., Sakhiya V., Miller I. (2017). Pregnancy outcomes in women on hemodialysis: a national survey. Clin Kidney J.

[22] Shah S., Christianson A.L., Thakar C.V., Kramer S., Meganathan K., Leonard A.C. (2020). Contraceptive use among women with end-stage kidney disease on dialysis in the United States. Kidney Med.

[23] Beral V., Doll R., Hermon C., Peto R., Reeves G., Cancer CGoESoO (2008). Ovarian cancer and oral contraceptives: collaborative reanalysis of data from 45 epidemiological studies including 23,257 women with ovarian cancer and 87,303 controls. Lancet.

[24] Hatzipetros I., Gocze P.M., Farkas B. (2013). Oral contraceptive pills as primary prevention for ovarian cancer: a systematic review and meta-analysis. Obstet Gynecol.

[25] Liu H., Yao J., Wang W., Zhang D. (2017). Association between duration of oral contraceptive use and risk of hypertension: A meta-analysis. J Clin Hypertens (Greenwich).

[26] Gierisch J.M., Coeytaux R.R., Urrutia R.P. (2013). Oral contraceptive use and risk of breast, cervical, colorectal, and endometrial cancers: a systematic review. Cancer Epidemiol Biomarkers Prev.

[27] Peragallo Urrutia R., Coeytaux R.R., McBroom A.J. (2013). Risk of acute thromboembolic events with oral contraceptive use: a systematic review and meta-analysis. Obstet Gynecol.

[28] Oedingen C., Scholz S., Razum O. (2018). Systematic review and meta-analysis of the association of combined oral contraceptives on the risk of venous thromboembolism: the role of the progestogen type and estrogen dose. Thromb Res.

[29] Lalude O.O. (2013). Risk of cardiovascular events with hormonal contraception: insights from the Danish cohort study. Curr Cardiol Rep.

[30] Plu-Bureau G., Maitrot-Mantelet L., Hugon-Rodin J., Canonico M. (2013). Hormonal contraceptives and venous thromboembolism: an epidemiological update. Best Pract Res Clin Endocrinol Metab.

[31] Koushik A., Grundy A., Abrahamowicz M. (2017). Hormonal and reproductive factors and the risk of ovarian cancer. Cancer Causes Control.

[32] Attini R., Cabiddu G., Montersino B. (2020). Contraception in chronic kidney disease: a best practice position statement by the Kidney and Pregnancy Group of the Italian Society of Nephrology. J Nephrol.

[33] Schürmann R., Blode H., Benda N., Cronin M., Küfner A. (2006). Effect of drospirenone on serum potassium and drospirenone pharmacokinetics in women with normal or impaired renal function. J Clin Pharmacol.

[34] Mantha S., Karp R., Raghavan V., Terrin N., Bauer K.A., Zwicker J.I. (2012). Assessing the risk of venous thromboembolic events in women taking progestin-only contraception: a meta-analysis. BMJ.

[35] Modesto W., Bahamondes M.V., Bahamondes L. (2015). Prevalence of low bone mass and osteoporosis in long-term users of the injectable contraceptive depot medroxyprogesterone acetate. J Womens Health (Larchmt).

[36] Moroni G., Doria A., Giglio E. (2016). Fetal outcome and recommendations of pregnancies in lupus nephritis in the 21st century. A prospective multicenter study. J Autoimmun.

[37] Bujold E., Roberge S., Lacasse Y. (2010). Prevention of preeclampsia and intrauterine growth restriction with aspirin started in early pregnancy: a meta-analysis. Obstet Gynecol.

[38] Wiles K., Chappell L., Clark K. (2019). Clinical practice guideline on pregnancy and renal disease. BMC Nephrol.

[39] Gomes S., Lopes C., Pinto E. (2016). Folate and folic acid in the periconceptional period: recommendations from official health organizations in thirty-six countries worldwide and WHO. Public Health Nutr.

[40] Hladunewich M.A. (2017). Chronic kidney disease and pregnancy. Semin Nephrol.

[41] El Sebaaly Z., Charpentier B., Snanoudj R. (2007). Fetal malformations associated with mycophenolate mofetil for lupus nephritis. Nephrol Dial Transplant.

[42] Götestam Skorpen C., Hoeltzenbein M., Tincani A. (2016). The EULAR points to consider for use of antirheumatic drugs before pregnancy, and during pregnancy and lactation. Ann Rheum Dis.

[43] Coscia L.A., Constantinescu S., Moritz M.J. (2010). Report from the National Transplantation Pregnancy Registry (NTPR): outcomes of pregnancy after transplantation. Clin Transpl.

[44] Østensen M., Lockshin M., Doria A. (2008). Update on safety during pregnancy of biological agents and some immunosuppressive anti-rheumatic drugs. Rheumatol (Oxf Engl).

[45] Perrotta K., Kiernan E., Bandoli G., Manaster R., Chambers C. (2021). Pregnancy outcomes following maternal treatment with rituximab prior to or during pregnancy: a case series. Rheumatol Adv Pract.

[46] Bullo M., Tschumi S., Bucher B.S., Bianchetti M.G., Simonetti G.D. (2012). Pregnancy outcome following exposure to angiotensin-converting enzyme inhibitors or angiotensin receptor antagonists: a systematic review. Hypertension.

[47] Fu J., Tomlinson G., Feig D.S. (2021). Increased risk of major congenital malformations in early pregnancy use of angiotensin-converting-enzyme inhibitors and angiotensin-receptor-blockers: a meta-analysis. Diabetes Metab Res Rev.

[48] Shotan A., Widerhorn J., Hurst A., Elkayam U. (1994). Risks of angiotensin-converting enzyme inhibition during pregnancy: experimental and clinical evidence, potential mechanisms, and recommendations for use. Am J Med.

[49] Malhamé I., Dong S., Syeda A. (2023). The use of loop diuretics in the context of hypertensive disorders of pregnancy: a systematic review and meta-analysis. J Hypertens.

[50] Mosley J.F., Smith L., Everton E., Fellner C. (2015). Sodium-glucose linked transporter 2 (SGLT2) inhibitors in the management of type-2 diabetes: a drug class overview. P T.

[51] Ofori B., Rey E., Bérard A. (2007). Risk of congenital anomalies in pregnant users of statin drugs. Br J Clin Pharmacol.

[52] FDA FDA requests removal of strongest warning against using cholesterol-lowering statins during pregnancy; still advises most pregnant patients should stop taking statins. https://www.fda.gov/drugs/drug-safety-and-availability/fda-requests-removal-strongest-warning-against-using-cholesterol-lowering-statins-during-pregnancy08/30/2021.

[53] Bateman B.T., Hernandez-Diaz S., Fischer M.A. (2015). Statins and congenital malformations: cohort study. BMJ.

[54] Chang J.C., Chen Y.J., Chen I.C., Lin W.S., Chen Y.M., Lin C.H. (2021). Perinatal outcomes after statin exposure during pregnancy. JAMA Netw Open.

[55] Richards T., Breymann C., Brookes M.J. (2021). Questions and answers on iron deficiency treatment selection and the use of intravenous iron in routine clinical practice. Ann Med.

[56] Tangren J., Nadel M., Hladunewich M.A. (2018). Pregnancy and end-stage renal disease. Blood Purif.

[57] Cunningham M.W., LaMarca B. (2018). Risk of cardiovascular disease, end-stage renal disease, and stroke in postpartum women and their fetuses after a hypertensive pregnancy. Am J Physiol Regul Integr Comp Physiol.

[58] (2013). Hypertension in pregnancy. Report of the American College of Obstetricians and Gynecologists’ Task Force on Hypertension in Pregnancy. Obstet Gynecol.

[59] Umesawa M., Kobashi G. (2017). Epidemiology of hypertensive disorders in pregnancy: prevalence, risk factors, predictors and prognosis. Hypertens Res.

[60] Ying W., Catov J.M., Ouyang P. (2018). Hypertensive disorders of pregnancy and future maternal cardiovascular risk. J Am Heart Assoc.

[61] Gupta S., Petras L., Tufail M.U., Rodriguez Salazar J.D., Jim B. (2023). Hypertension in pregnancy: what we now know. Curr Opin Nephrol Hypertens.

[62] Kattah A. (2020). Preeclampsia and kidney disease: deciphering cause and effect. Curr Hypertens Rep.

[63] Zeisler H., Llurba E., Chantraine F. (2016). Predictive value of the sFlt-1:PlGF ratio in women with suspected preeclampsia. N Engl J Med.

[64] Magee L.A., von Dadelszen P., Rey E. (2015). Less-tight versus tight control of hypertension in pregnancy. N Engl J Med.

[65] Magee L.A., von Dadelszen P. (2022). Treatment for mild chronic hypertension during pregnancy. N Engl J Med.

[66] Toma H., Tanabe K., Tokumoto T., Kobayashi C., Yagisawa T. (1999). Pregnancy in women receiving renal dialysis or transplantation in Japan: a nationwide survey. Nephrol Dial Transplant.

[67] Luders C., Castro M.C., Titan S.M. (2010). Obstetric outcome in pregnant women on long-term dialysis: a case series. Am J Kidney Dis.

[68] Hladunewich M.A., Hou S., Odutayo A. (2014). Intensive hemodialysis associates with improved pregnancy outcomes: a Canadian and United States cohort comparison. J Am Soc Nephrol.

[69] Redrow M., Cherem L., Elliott J. (1988). Dialysis in the management of pregnant patients with renal insufficiency. Med (Baltimore).

[70] Veríssimo R., Nogueira E., Bernardo J. (2022). Pregnancy in a woman undergoing peritoneal dialysis: Management and dialysis options. Clin Nephrol Case Stud.

[71] Lim T.S., Shanmuganathan M., Wong I., Goh B.L. (2017). Successful multigravid pregnancy in a 42-year-old patient on continuous ambulatory peritoneal dialysis and a review of the literature. BMC Nephrol.

[72] Batarse R.R., Steiger R.M., Guest S. (2015). Peritoneal dialysis prescription during the third trimester of pregnancy. Perit Dial Int.

[73] Smith W.T., Darbari S., Kwan M., O Reilly-Green C., Devita M.V. (2005). Pregnancy in peritoneal dialysis: a case report and review of adequacy and outcomes. Int Urol Nephrol.

[74] Cabiddu G., Castellino S., Gernone G. (2016). A best practice position statement on pregnancy in chronic kidney disease: the Italian Study Group on Kidney and Pregnancy. J Nephrol.

[75] Okundaye I., Hou S. (1996). Management of pregnancy in women undergoing continuous ambulatory peritoneal dialysis. Adv Perit Dial.

[76] Okundaye I., Abrinko P., Hou S. (1998). Registry of pregnancy in dialysis patients. Am J Kidney Dis.

[77] Oliverio A.L., Hladunewich M.A. (2020). End-stage kidney disease and dialysis in pregnancy. Adv Chronic Kidney Dis.

[78] Manisco G., Potì M., Maggiulli G., Di Tullio M., Losappio V., Vernaglione L. (2015). Pregnancy in end-stage renal disease patients on dialysis: how to achieve a successful delivery. Clin Kidney J.

[79] Barua M., Hladunewich M., Keunen J., Pierratos A., McFarlane P., Sood M., Chan C.T. (2008). Successful pregnancies on nocturnal home hemodialysis. Clin J Am Soc Nephrol.

